# How to report neurotechnology and artificial intelligence studies in epilepsy: Peer‐review‐inspired recommendations

**DOI:** 10.1002/epi4.70194

**Published:** 2025-11-27

**Authors:** Pedro F. Viana, Matthew McWilliam, Andrea Biondi, Jesus Servando Medel‐Matus, Naoto Kuroda, Giulia Sofia Cereda, Aristea S. Galanopoulou

**Affiliations:** ^1^ Institute of Psychiatry, Psychology and Neuroscience King's College London London UK; ^2^ Epilepsy Centre King's College Hospital NHS Foundation Trust London UK; ^3^ Centro de Estudos Egas Moniz, Faculdade de Medicina Universidade de Lisboa Lisbon Portugal; ^4^ Department of Pediatrics, Neurology Division David Geffen School of Medicine at University of California Los Angeles Los Angeles California USA; ^5^ Department of Pediatrics Wayne State University Detroit Michigan USA; ^6^ Department of Epileptology Tohoku University Graduate School of Medicine Sendai Japan; ^7^ Epilepsy Unit Fondazione IRCCS Istituto Neurologico Carlo Besta Milan Italy; ^8^ Saul R. Korey Department of Neurology, Isabelle Rapin Division of Child Neurology, Dominick P. Purpura Department of Neuroscience, Laboratory of Developmental Epilepsy Albert Einstein College of Medicine Bronx New York USA

**Keywords:** artificial intelligence, epilepsy, machine learning, neurotechnology, standards

## Abstract

**Objective:**

The integration of neurotechnology and artificial intelligence (AI) in epilepsy research has led to significant advancements in diagnosis, monitoring, and treatment. However, the impact of these innovations is often diminished by inadequate and inaccurate reporting, limiting their reproducibility and implementation. This study aimed to identify common peer review concerns and develop reporting recommendations specific to neurotechnology and AI studies in epilepsy.

**Methods:**

We conducted a qualitative analysis of peer review comments from original research article submissions to *Epilepsia Open* over a 2‐year period (September 2021–August 2023). We selected manuscripts that focused on neurotechnology or AI applications in epilepsy, excluding those using standard clinical technologies or conventional statistical analyses. Reviewer comments were classified using a validated checklist, categorizing issues into themes and subthemes. Based on recurrent peer review concerns, we developed a set of reporting recommendations for neurotechnology and AI studies.

**Results:**

Among 329 manuscripts sent for peer review, 67 were classified as neurotechnology or AI studies and included in the analysis. These studies predominantly involved advanced neuroimaging analysis, advanced electroencephalography (EEG) analysis, and neuromodulation systems. Reviewer comments were primarily focused on study methodology (37%), manuscript presentation (19%), discussion (17%), and results (12%). Based on peer review comments, we formulated reporting recommendations, hoping to enhance study transparency, methodological rigor, and reproducibility.

**Significance:**

Our reporting recommendations address key concerns raised during peer review, providing guidance to authors and reviewers to improve the quality and clarity of neurotechnology and AI research in epilepsy. These recommendations complement existing reporting standards and contribute to the advancement of robust and impactful research in the field.

**Plain Language Summary:**

We studied how researchers report studies on neurotechnology and AI in epilepsy. Many studies face problems during peer review, such as unclear methods, weak study rationale, and errors in statistics or citations. We analyzed reviewer feedback and created recommendations to improve how these studies are reported. Our goal is to help researchers develop and present their work more clearly and accurately, making it easier for others to understand and build upon their findings. This can lead to better use of AI and neurotechnology in epilepsy research and care.

**Key points**

- Peer review of neurotechnology and AI epilepsy studies revealed frequent methodological and reporting issues.
- Reviewer concerns mainly involved study methodology, presentation, discussion, and results.
- Reporting recommendations were developed to improve transparency, rigor, and reproducibility in these studies.
- The proposed recommendations aim to help authors and reviewers enhance clarity and impact in AI and neurotechnology research.

## 1 INTRODUCTION

Rapid progress in cutting‐edge sensing technologies, advanced materials, and artificial intelligence (AI) provides ever increasing possibilities to address difficult societal problems, including in healthcare. Within the field of epilepsy, the integration of neurotechnology and AI offers a wide range of opportunities to improve diagnosis, phenotyping, decision support, monitoring, and treatment recommendations.^1^

The availability of large and complex data sets, including neurophysiology,^2^, ^3^ neuroimaging,^4^, ^5^ wearable^6^ and multiomics^7^ data, is well suited to the use of data‐driven AI techniques, which are seeing widespread application in challenging areas in epilepsy. Examples include automating and accelerating diagnosis,^8^ automated seizure detection,^9^ seizure forecasting,^10^ epilepsy phenotyping (e.g., lateralization/localization of the epileptogenic zone),^11^ surgical management and prediction of surgical outcome,^12^ prediction of drug‐resistance, and of treatment response.^1^, ^13^, ^14^

In parallel, the development of new technological solutions to monitor and treat epilepsy, including neuromodulation,^15^ wearable sensors,^16^ mobile EEG systems,^17^ and smartphone applications,^18^ further provides high volume and high velocity data, and hence are themselves drivers of more sophisticated data‐driven analytical techniques.

Despite the increasing number of studies reporting on these innovations, the impact of research findings is often diminished due to several factors, including inadequate and inaccurate reporting. Inaccurate reporting impairs the ability to critically appraise study design and methods, have confidence in the findings and further evaluate or implement a new solution. This has led to the development of specific reporting standards that have evolved to address the increasing complexity of AI studies, such as the *Consolidated Standards of Reporting Trials‐Artificial Intelligence* (CONSORT‐AI)^19^ and the *Transparent Reporting of a multivariable prediction model for Individual Prognosis Or Diagnosis* statement (TRIPOD‐AI).^20^ Additionally, efforts have been made to establish common data elements for both preclinical and clinical research to enhance standardization and data comparability across studies.^21^ For an overview of such resources, see Table 1.

**TABLE 1:** Selected list of available reporting recommendations on neurotechnology and artificial intelligence (AI) in medicine, neurology, and epilepsy.

Resource	Scope
TRIPOD+AI^20^	Standards to report development and evaluation of a prediction model, including using AI
SPIRIT‐AI^22^	Standards to design protocols for clinical trials evaluating an AI‐based intervention
CONSORT‐AI^19^	Standards to report clinical trials evaluating an AI‐based intervention
CLAIM^23^	Standards to report medical imaging studies using AI
STRATOS Initiative^24^	Guidance for design and analysis of observational studies, including high‐dimensional data
Harte‐Hargrove et al. (2018)^21^	Common data elements (CDEs) for preclinical epilepsy research
Goldenholz et al. (2018)^25^	Common data elements for epilepsy mobile health systems
Beniczky et al. (2018)^26^	Standards for testing and clinical validation of seizure detection devices
Bruno et al. (2020)^6^	Practical recommendations for wearable device research in epilepsy
Chiang et al. (2021)^27^	Ethical guidelines for conducting AI research in Neurology
Speiser et al. (2024)^28^	Common critiques and recommendations for studies in Neurology using machine‐learning methods
Goldenholz et al. (2023)^29^	Common pitfalls and recommendations on using machine learning for post‐surgical outcome prediction

In this report, we aimed to complement available reporting standards by adapting them to the context of neurotechnology and AI in epilepsy, and inspired by peer review. Peer review is central to scientific quality assurance, offering critical assessment that can identify methodological weaknesses, as well as other recurring issues.^30^, ^31^ We identified common pitfalls and concerns raised by peer review comments in an epilepsy journal, and developed a set of specific reporting recommendations on neurotechnology and AI in epilepsy. These recommendations can help reviewers assess the quality of emerging technologies more effectively and guide future authors in improving study design and the overall quality of submitted manuscripts.

## 2 METHODS

We analyzed peer review comments from original research articles (full‐length and short) manuscript submissions to *Epilepsia Open*, over a 2‐year period (September 1st, 2021–August 31st, 2023). We included manuscripts sent for peer review and those with a final decision reached at the time of data collection (~90% of submissions).

**FIGURE 1:**
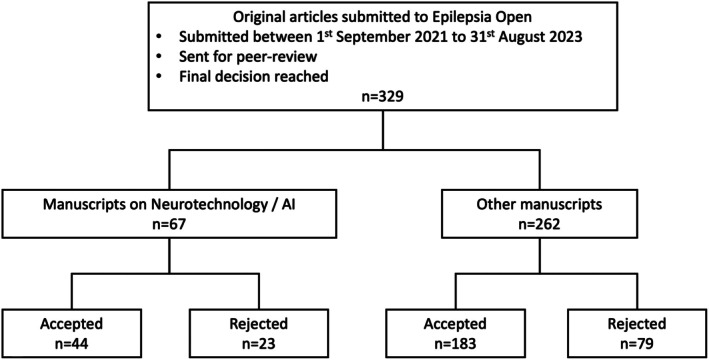
Overview of the submissions included in the qualitative analysis.

We manually reviewed manuscripts and selected those reporting on the use of neurotechnology or AI in epilepsy. As there is a lack of a formal definition of neurotechnology, we used an inclusive approach, including papers reporting on neural sensing devices, advanced neuroimaging and neurophysiology techniques, novel neuromodulation devices and digital health technologies, and excluded papers using standard, clinically approved technological solutions. We also included studies using advanced statistical analysis and machine learning, while excluding clinical cohort descriptions or studies using standard statistical analysis. A flowchart of included manuscripts is shown in Figure 1.

Data collected included the manuscript title, abstract, and keywords; initial decision letters with reviewer comments; final decision reached (accepted vs. rejected after final review) and number of reviewers per manuscript. A panel of three raters (P.F.V., M.M., A.B.) qualitatively assessed peer reviewer comments. Panel members were blinded for author names and affiliations, as well as for the final manuscript decision. Each comment or statement was classified using a previously validated checklist (Table S1).^32^ This checklist classifies each comment into a main theme (e.g., background, methods), and subsequently into a subtheme (e.g., within the Methods theme: poor experimental design; methods inadequately reported; inappropriate statistical analysis). The three raters independently classified 25–27 manuscripts, with ~20% (*n* = 5 papers) overlap to assess inter‐rater agreement (Table S2). On the manuscripts with overlap, a final classification was reached via consensus. Comment classification was performed with the open‐source qualitative data analysis tool Taguette.^33^ We explored which types of comments were associated with manuscript acceptance rate, using Wilcoxon Rank‐sum tests.

Next, the panel collected common concerns raised by reviewers, within each checklist item, and devised a set of reporting recommendations, which were then jointly discussed and shared for approval with all co‐authors (Table 3). Each reporting recommendation is coupled with example pitfalls within the field of epilepsy, and also includes example reviewer comments (see Table S3).

## 3 RESULTS

**FIGURE 2:**
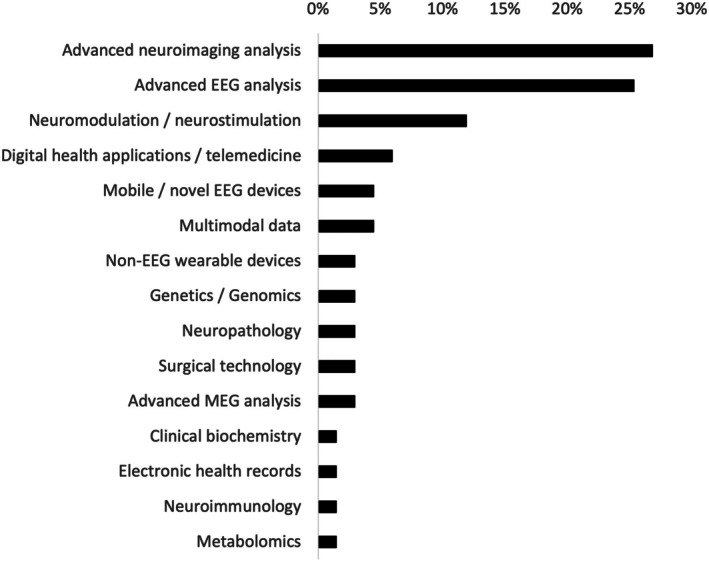
Type of technology used per included study.

**FIGURE 3:**
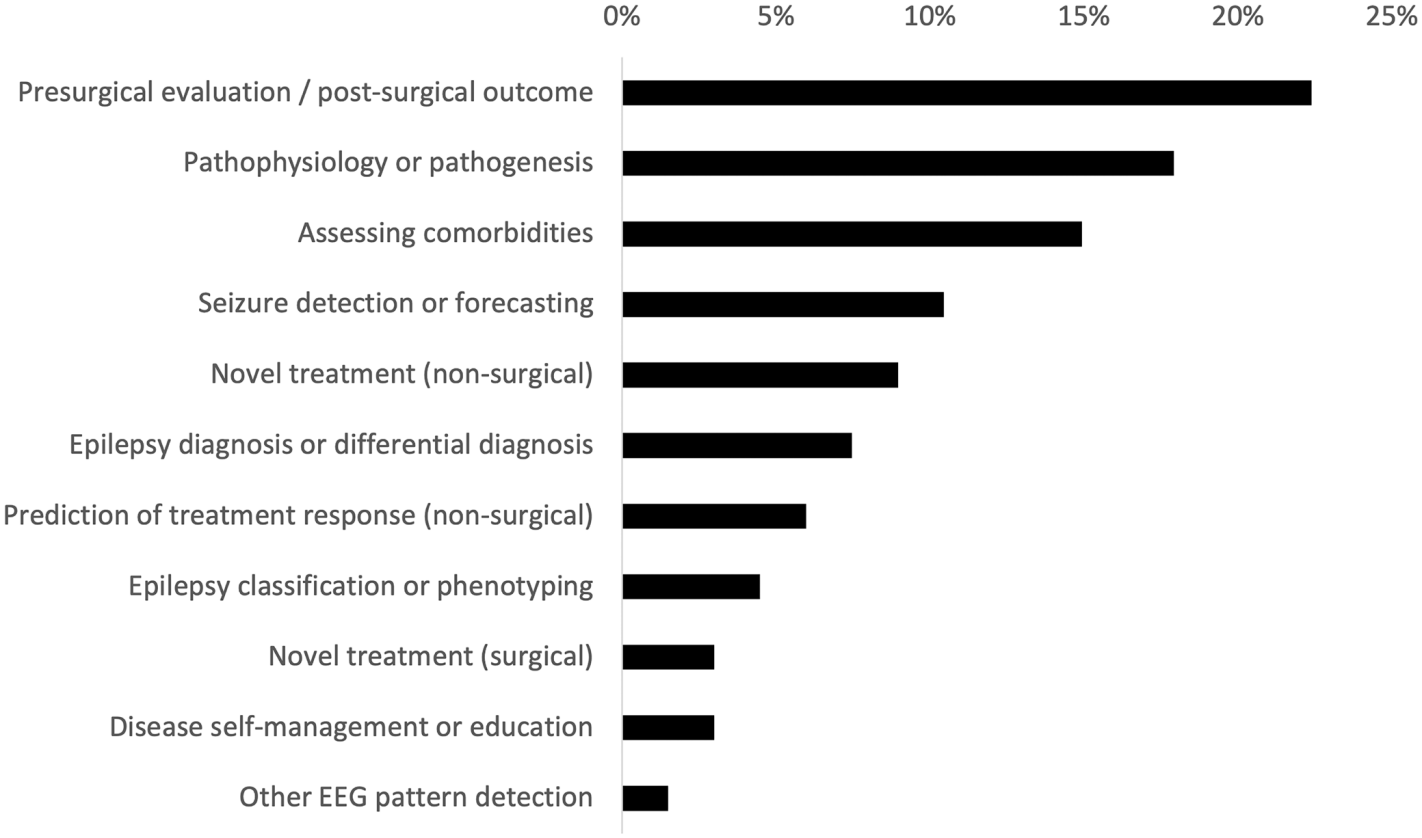
Clinical applications aimed at per included study.

After identifying all submissions within a 2‐year period that were sent for peer review (*n* = 329), a total of 67 manuscripts were classified as Neurotechnology or AI studies and were included in the qualitative analysis (Figure 1). Most included studies were those using advanced neuroimaging or EEG analysis or reporting on neuromodulation/neurostimulation systems (Figure 2). Most studies were directed at presurgical investigation or prediction of post‐operative outcomes, at improving understanding of the pathophysiology or pathogenesis of epilepsy, followed by assessing epilepsy comorbidities and seizure detection or forecasting (Figure 3).

There was no statistically significant difference in the acceptance rate between Neurotechnology and AI studies and other studies (Figure S1—65.7% vs. 69.8%, Chi‐squared test, *p* = 0.51). There was also no statistically significant difference in the number of reviewers between the two types of studies (Figure S2—Chi‐squared test, *p* = 0.54).

Regarding classification of reviewer comments, a total of 1059 comments were distinctively classified into themes and subthemes (average 15.8 comments per manuscript). The overall percentage agreement among the three raters was high (88%), with some discrepancies noted within the background, methods, results, and discussion themes and respective sub‐themes (Table S2). Reviewer comments were mostly addressed at the study methods (37%), followed by manuscript presentation (19%), discussion (17%), and results (12%).

On univariate analysis comparing accepted versus rejected manuscripts, lack of study novelty, poor study rationale, inappropriate statistical analysis, inappropriate conclusions, errors in the abstract, and citation errors showed a trend toward association with article rejection; however, this was not statistically significant after correction for false discovery rate (Table 2).

**TABLE 2:** Themes and sub‐themes associated with article acceptance or rejection (average number of comments per reviewer per manuscript).

Section	Comment	All (*n* = 67)	Accepted (*n* = 44)	Rejected (*n* = 23)	*p*‐value^a^	Adjusted *p*‐value^b^
		Mean (SD)	Mean (SD)	Mean (SD)		
Importance	Limited clinical relevance	0.07 (0.31)	0.06 (0.22)	0.11 (0.43)	0.812	0.886
Originality	Lack of novelty	0.17 (0.4)	0.08 (0.22)	0.33 (0.58)	**0.020**	0.115
Abstract	Errors abstract	0.02 (0.11)	0 (0)	0.07 (0.17)	**0.018**	0.115
	Abstract does not correctly reflect paper	0.17 (0.33)	0.13 (0.27)	0.25 (0.42)	0.216	0.479
Background	Incorrect background information	0.22 (0.36)	0.19 (0.35)	0.28 (0.39)	0.229	0.479
	Poor study rationale	0.12 (0.28)	0.08 (0.27)	0.19 (0.29)	**0.037**	0.146
Methods	Methods inadequately reported	1.88 (2.04)	1.82 (2.25)	2 (1.64)	0.367	0.529
	Poor experimental design	1.09 (0.95)	0.91 (0.77)	1.41 (1.15)	0.085	0.279
	Statistical analysis inappropriate	0.41 (0.61)	0.26 (0.4)	0.68 (0.81)	**0.008**	0.115
Results	Flow of participants unclear	0.1 (0.24)	0.07 (0.21)	0.16 (0.29)	0.143	0.365
	Outcome data incomplete	0.55 (0.8)	0.54 (0.78)	0.56 (0.84)	0.856	0.886
Discussion	Conclusions inappropriate	0.52 (0.8)	0.4 (0.8)	0.74 (0.78)	**0.013**	0.115
	Literature insufficiently discussed	0.2 (0.39)	0.22 (0.44)	0.16 (0.29)	0.880	0.886
	Limitations insufficiently discussed	0.36 (0.61)	0.44 (0.7)	0.23 (0.38)	0.348	0.529
	Results inadequately discussed	0.42 (0.57)	0.43 (0.58)	0.4 (0.57)	0.886	0.886
References	Citation errors	0.06 (0.18)	0.02 (0.11)	0.12 (0.26)	**0.038**	0.146
	Omitted relevant citations	0.28 (0.7)	0.36 (0.84)	0.15 (0.32)	0.349	0.529
Presentation	Inaccurate tables or figures	0.6 (0.71)	0.58 (0.8)	0.62 (0.55)	0.368	0.529
	Incorrect title	0.09 (0.25)	0.08 (0.24)	0.11 (0.26)	0.575	0.778
	Poor writing	1.04 (1.38)	0.88 (1.38)	1.33 (1.37)	0.134	0.365
Ethics	Conflict of interest concerns	0.03 (0.15)	0.01 (0.08)	0.07 (0.23)	0.250	0.479
	Other ethical concerns	0.02 (0.14)	0.01 (0.08)	0.04 (0.21)	0.663	0.847
Other	Other	0.14 (0.38)	0.14 (0.39)	0.13 (0.38)	0.751	0.886

*Note*: Significant *p*‐values in bold.

^a^ Wilcoxon rank‐sum tests.

^b^ Benjamini‐Hochberg false discovery rate correction.

After peer review comment analysis, a set of reporting recommendations for neurotechnology and AI studies in epilepsy was developed (Table 3). Each recommendation is organized by an overarching theme within a corresponding manuscript section, although several themes can span multiple sections. To illustrate common issues, each recommendation is accompanied by hypothetical pitfall case scenarios specific to epilepsy research. In parallel with the reviewer comments, most recommendations concern the study methodology.

**TABLE 3:** Recommendations for reporting neurotechnology and AI studies in epilepsy.

Theme	Recommendation(s)	Example pitfalls in epilepsy^a^
*Background and objectives*		
Justify the use of the new technology or AI	Justify the choice of the new technology or AI method. Describe how it adds to the existing clinical workflow or pathway. Compare it with current existing similar solutions. Simpler methods are preferred if performance is comparable. Avoid using new technology or AI solely for novelty.	An AI‐based seizure detection algorithm requiring high computational resources has similar performance a simpler algorithm based on a small number of features.
Align study goals with the analysis	Clearly define study objectives and ensure analysis aligns with these goals. Provide rationale for the study hypotheses.	A study on a seizure detection system aimed at providing a real‐time alarm to patients does not report on the detection latency of their algorithm.
*Methods*		
Study population/data source	Describe inclusion and exclusion criteria for participants, screening method(s). Describe all data sources used for model training and testing, including type, representativeness, and rationale. Consider selection bias.	A post‐operative outcome algorithm is trained on a single centre with no experience in a particular surgical technique. Therefore, the model is unlikely to generalize.
Participant/data flow	Report participant flow, follow‐up or loss to follow‐up. Describe handling of missing or poor‐quality data, including imputation or exclusion procedures.	In a neurostimulation trial, patients with device complications or non‐effectiveness may drop out early and less likely attend monitoring visits, leading to an inflated estimation of device effectiveness.
Description of the technology	Describe the technology fairly for readers who might not be familiar with it.	A paper describes a “wearable seizure detector” but does not clarify that the device only relies on accelerometery and is only useful to detect major motor seizures.
Control group or intervention	Aim to isolate the effect of the new technology or method, by comparing with a control group or intervention.	A novel neurostimulation device is associated with a reduction in seizure frequency, however, there is no sham control period to isolate the effect of stimulation from the effect of surgery.
Data collection and preprocessing	Describe them enough to allow replication. Justify the choice of preprocessing steps or stimulation parameters with previous literature or available recommendations. EEG studies: e.g., sampling rate, filtering, referencing, montages, artifact rejection, epoching, electrode material, data normalization. Neuroimaging studies: e.g. type of scanner, consistency across patients, timing/duration of tracer uptake, circumstances of scanning.	In a neuroimaging AI lesion detection study, two centers use different scanner. One center has disproportionally more lesional cases than the other. The model learns scanner‐specific features, not pathology. A study aimed at detecting HFOs uses inappropriate high pass filtering.
Data annotation	Use qualified data annotators or raters, ideally blinded for study groups or outcomes. Use standard or validated data collection instruments. Acknowledge limitations in reliability of data annotations	A non‐qualified PhD student reviews EEG data to annotate seizure patterns and semiology, leading to many falsely annotated events (artifacts) as seizures. A study uses information from a seizure diary smartphone application—the diagnosis of epilepsy is not confirmed by experts.
Separate training from testing data sets	Use separate data sets for training and testing. Describe partitioning, cross‐validation, or bootstrapping methods. Only tune model parameters on the training or validation data sets, not on the testing data set.	In a seizure forecasting study, the calculation of the predictor features is influenced by future datapoints.
Sample size	Perform and report sample size estimation/power calculation, adequate for the objectives of the study, or justify low sample size, or use appropriate methods to circumvent the small sample size (e.g., transfer learning from another data set).	In a neuroimaging study investigating lesion detection in epilepsy, training a neural network with thousands of parameters on only a small cohort of patients can lead to overfitting, causing artificially high performance on the training data but poor generalization to new patients.
Class imbalance	Address or correct for class imbalance. Consider balanced sampling when splitting data into training and testing.	In a seizure detection study, an algorithm that classifies all events as “interictal” may have 99% accuracy, simply because the ratio of interictal to ictal samples is large.
Model specification	Describe the AI models—input structure, architecture, output structure. Specify model type, approach, and all steps in building, including hyperparameter tuning, parameter handling, and internal validation.	A model claims to predict seizures from EEG but does not specify whether the input is raw EEG segments, power spectral features, or time‐of‐day information.
Reference standard	Use an appropriate clinical reference standard when comparing with your solution. Comparison between the proposed solution and clinical standard should be direct and fair (e.g., manual vs. automated methods, old vs. new technology).	A seizure detection study compares an automated seizure detection algorithm with manual review. However, experts only review the algorithm's detections, and therefore there is no assessment of false negatives. A seizure detection study compares a detection algorithm with video analysis, including for seizures with no clear clinical manifestations.
Multiple comparisons	Address for multiple comparisons and false discovery rate.	A study of high‐density EEG spectral analysis computes spectral power in four frequency bands, per channel, leading to hundreds of statistical tests.
*Results*		
Model evaluation and comparison	Use clinically relevant performance metrics. Report effect sizes and measures of dispersion (e.g., confidence intervals) around performance. Consider performing feature importance analyses. Discuss proxy outcomes and limitations.	In a post‐surgical outcome prediction study, only the AUC is provided, whilst the negative predictive values (NPV) would likely have more clinical value.
Model fairness	Consider whether the model is fair or whether there is a risk of perpetuating existing biases in the data set.	A post‐operative outcome model uses age as a major determinant in outcome, potentially leading to self‐fulfilling prophecy of denying surgery to older individuals.
Technology acceptability and safety	Describe how relevant stakeholders (patients, clinicians, caregivers) were involved in technology and AI design and conceptualization. Report acceptability and usability of the new technology or method, e.g., adherence, app usage. Report safety of using the technology.	A wearable device company develops an epilepsy monitoring device with a poor design, which leads to a low acceptability and high attrition rate.
Confounding	Address or acknowledge confounding.	In an EEG study developing an AI algorithm to diagnose epilepsy, using patient recordings on anti‐seizure medication while controls are medication‐free may cause the model to learn drug‐induced EEG changes rather than true disease‐related features.
*Discussion*		
Clinical applicability and integration	Describe the technology integration into clinical workflow, including technical requirements, human interaction, and user expertise. Discuss technology limitations, e.g., cost, technical support, data security, limited adherence, device deficiency. Describe the target population for which the technology could be useful. Provide future directions for further research and validation of the technology.	A seizure monitoring device placed under the mattress is only useful when patients are in bed. A neuroimaging automated lesion detection solution uses a research‐grade, non‐medically approved preprocessing toolbox.
*Presentation*		
Terminology	Use updated, accepted or widely used terminology. Refer to glossaries, recent guidelines, ILAE position papers, and journal instructions for authors. Avoid technical jargon and adapt the language to the target journal. Proof‐reading by a native speaker is recommended.	In a study on automated EEG seizure detection, the authors use the term “epileptic events” interchangeably with “seizures” and “spikes,” without defining each; this inconsistency confuses readers about whether the model detects ictal episodes, interictal discharges, or both.
*Ethics*		
Fairness	Avoid writing with bias, e.g., commercial bias. Be fair in the interpretation of your results.	Authors affiliated with a neurotechnology company emphasize the “superior performance” of their proprietary software without disclosing that competing open‐source models were not evaluated under identical conditions.
Data availability and code availability	Ensure transparency and reproducibility by sharing code, models, and documentation.	In an AI study on seizure detection, not sharing preprocessing code led to an undetected labelling error where postictal segments were included as seizures, artificially inflating model performance.

^a^ These are hypothetical examples and not directly related to any real examples in the data set. For anonymized peer‐review comments relevant to each recommendation, see Table S3.

## 4 DISCUSSION

Our study highlights recurrent challenges in the reporting of neurotechnology and AI studies in epilepsy, as identified through peer review comments. By systematically categorizing and analyzing reviewer concerns, we identified common critical areas that require improvement, including study methodology, manuscript presentation, and interpretation/discussion of results. Based on these findings, we formulated a set of reporting recommendations aimed at enhancing transparency, methodological rigor, and reproducibility in the field.

### 4.1 Background and objectives section

Reviewers frequently criticized manuscripts for lacking a clear justification for the use of AI or of novel technologies. Several studies failed to explain how their approach improved existing clinical practice methods or workflows. Technologies are often applied primarily for novelty's sake, rather than because there is a demonstrated need or added value.^34^ Researchers should also provide a clear rationale for their study hypotheses, any unmet needs fulfilled with the current study, and ensure that their analyses align with research objectives.^28^

### 4.2 Methodology section

The most common criticism (~40% of all comments) related to the inadequate reporting of study methodology. Given the complexity of neurotechnology and AI‐driven studies, precise descriptions of data sets, analysis pipelines, model specifications, and statistical analysis are essential for reproducibility.

Despite the advances in machine‐learning methodology, fundamental issues related to data quality and representativeness remain major sources of bias. The principle of “*garbage in, garbage out*” continues to apply, as no algorithm can compensate for biased, incomplete, or poorly curated data.^28^ Many studies fail to comprehensively report eligibility criteria or to justify the selection of their data sources. Patients who enroll in neurotechnology research are often highly selected, for instance, recruited from tertiary epilepsy centers and thus may not represent the broader epilepsy population. Such selection biases limit the generalizability of model predictions. Incomplete reporting of participant flow, follow‐up, or attrition introduces uncertainty about potential losses of certain subgroups, such as patients with poorer outcomes or device complications. Missing data present additional challenges. Excluding cases with missing values can exacerbate bias if data are not missing at random,^28^ whereas inappropriate imputation may introduce artificial patterns. Authors should therefore describe missing data explicitly, justify their handling approach (e.g., imputation, exclusion, or model‐based correction), and discuss the potential implications for bias and poor model generalization.

Another commonly criticized aspect of data quality concerns the accuracy of data annotations. Curating and annotating large, high‐velocity data sets is both time‐consuming and resource‐intensive for clinicians and researchers. Biased or inaccurate annotations (for example, non‐specialized raters erroneously labeling artifacts as seizures), can amplify existing biases in the data. High inter‐rater variability such as in EEG review underscores the importance of using multiple expert raters who are ideally blinded to study groups or outcomes, as well as using standardized definition criteria^35^ and annotation instruments.^36^

Data preprocessing is a critical step. Improper filtering, normalization, or artifact removal can introduce biases and inflate model performance. The use of widely varied, often center‐specific preprocessing pipelines hinders reproducibility across studies.^37^ As such, a collaborative effort is needed to standardize preprocessing practices in epilepsy research. The choice of preprocessing steps should be carefully justified, ideally based on established consensus, published recommendations, or prior literature. Whenever possible, preprocessing code should be made publicly available to enable independent verification and replication.

Many machine‐learning models are highly data‐dependent, requiring large numbers of samples relative to the number of model parameters to achieve reliable performance. Training a model on small cohorts, particularly using high‐dimensional genomic, neuroimaging or EEG data, often leads to overfitting, with inflated performance on the training data and poor generalization to new cohorts.^38^ To mitigate this, data sets must be large enough, and split into separate training/validation and testing sets, ensuring that test data remain completely unseen during model development, including during parameter tuning. Care should also be taken to account for class imbalance commonly seen in epilepsy research data (e.g., in seizure detection studies, when interictal periods vastly outnumber ictal events).^28^, ^29^ The availability of well‐curated, large shared data sets for public analysis and algorithm development has proven valuable, such as in automated seizure detection or forecasting.^2^, ^39^, ^40^ In cases where data are scarce, simpler statistical imputation, small feature models, or transfer learning are options to consider.^41^, ^42^

### 4.3 Results section

The measures of performance to preferably report for a model are highly dependent on the use case. For example, in post‐operative outcome studies, reporting only the area under the curve may have limited clinical value, whereas reporting the negative predictive value (i.e., the probability that patients predicted to have a poor outcome truly do) can be more meaningful for clinical decision making.^29^ Another example is the reporting of accuracy in the context of a highly unbalanced data set, such as in seizure detection. An algorithm which always classifies “no seizure” could achieve an accuracy of 99.9%, simply reflecting the predominance of interictal data.^6^

Second, effect sizes and measures of dispersion, such as confidence intervals, should be reported to contextualize model reliability, and feature importance analyses can help interpret model explainability.

Equally important is reporting the acceptability and safety of the technology among relevant stakeholders, including patients, clinicians, and caregivers.^16^ Usability metrics, adherence data, and adverse events should be documented, and any limitations in integration into clinical workflows should be discussed to ensure that the technology can be safely and effectively implemented.

### 4.4 Discussion section

For neurotechnology and AI interventions to be clinically useful, their integration into routine workflows must be carefully considered. Technical requirements, user expertise, and the nature of human interaction with the technology can limit applicability; for example, a seizure monitoring device placed under a mattress only captures seizures while the patient is in bed, and some implantable devices may not be MRI‐compatible. Cost, technical support, data security, and device reliability also constrain broader adoption, particularly for invasive approaches such as stereo‐EEG or research‐grade neuroimaging tools that are not clinically approved. Authors should clearly define the target population for which their technology is intended and discuss limitations that may affect generalizability. Future studies should focus on validating these tools in real‐world clinical settings, assessing usability, safety, and impact on patient outcomes.

### 4.5 Presentation

The second‐most common theme of concern to reviewers referred to manuscript presentation. Nearly 20% of reviewer comments pointed to issues related to manuscript structure, language clarity, and coherence. Reviewers noted inconsistencies in terminology, vague descriptions of findings, or, on the other hand, the use of overly technical jargon that hindered accessibility to a broader clinical audience. To address this, our recommendations include proofreading by a native speaker, consistent use of terminology, and avoiding redundant or overly general statements. We believe this is particularly important in the multidisciplinary context of neurotechnology and AI research, where differences in language between disciplines (e.g., engineers vs. clinicians) can exacerbate communication challenges.

### 4.6 Ethics

Ethical transparency and fairness are critical in neurotechnology and AI research. Authors should avoid introducing bias, including commercial bias, and provide balanced interpretations of their results; for example, emphasizing proprietary software performance without fair comparison to alternative methods can mislead readers. Once again, transparency in data and code availability is equally important to ensure reproducibility and allow independent verification of results. As previously mentioned, care should be taken to avoid self‐fulfilling prophecies related to disproportionately represented or vulnerable subgroups within training cohorts.^27^

### 4.7 Limitations of our analysis

While our study reports issues commonly raised during peer review of neurotechnology or AI manuscripts, it is difficult to ascertain which among the identified weaknesses contributed more to the rejection of these articles. In general, features that can be corrected with a revision carry less weight in rejecting these submissions, provided that there is additional merit and potential significance or novelty in the study. These include the manuscript presentation, discussion, references, or background weaknesses. Weaknesses, however, that are perceived as fatal or requiring extensive re‐writing and re‐analysis of the data are more likely to lead to rejection or rejection with an offer to resubmit. Examples include the inappropriate use of analysis methods, poor experimental design, bias in data collection or inadequate revision that fails to address reviewers' concerns.

## 5 CONCLUSIONS

In conclusion, our reporting recommendations provide a structured approach to improving the quality and transparency of neurotechnology and AI research in epilepsy. By addressing the most common peer review concerns, these guidelines can serve as a valuable resource for authors, reviewers, and journal editors. Implementing these recommendations can not only enhance the rigor of individual studies but also contribute to the broader goal of advancing reproducible and clinically impactful research in epilepsy.

## AUTHOR CONTRIBUTIONS

Study conception and design: P.F.V., J.S.M.‐M., G.S.C., N.K., A.S.G.; Data annotation and analysis: P.F.V., A.B., M.M.; first draft: P.F.V.; revisions: all authors.

## FUNDING INFORMATION

A.S.G. acknowledges grant support by NINDS U54 NS100064, NINDS R01 NS127524, a pilot grant from NICHD center grant (P50 HD105352) for the Rose F. Kennedy Intellectual and Developmental Disabilities Research Center (RFK‐IDDRC), U.S. Department of Defense (W81XWH‐22‐1‐0510, W81XWH‐22‐1‐0210, HT9425‐24‐1‐0134), the Heffer Family and the Segal Family Foundations, the Isabelle Rapin Family Foundation, and the Abbe Goldstein/Joshua Lurie and Laurie Marsh/Dan Levitz families. P.F.V. is supported by the NIHR Development and Skills Enhancement Award (NIHR NIHR305718). The views expressed are those of the author(s) and not necessarily those of the NIHR or the Department of Health and Social Care.

## CONFLICT OF INTEREST STATEMENT

A.S.G. was the editor‐in‐chief of *Epilepsia Open until August 2024* and is also an associate editor of *Neurobiology of Disease*, receives royalties from Elsevier (publications, journal editorial board participation), Wolters Kluwer Publishing (publications), and Medlink (publications), and an honorarium for her role as physician consultant for Synergy Medical Solutions. P.F.V. has received travel and consultancy fees from UNEEG Medical A/S; travel fees from Angelini Pharma, speaker fees from Eisai Pharmaceuticals. We confirm that we have read the Journal's position on issues involved in ethical publication and affirm that this report is consistent with those guidelines.

## Supporting information


**Table S1.** Peer reviewer comment classification checklist (adapted from van Lent et al., BMJ Open 2015).
**Figure S1.** Number of accepted versus rejected manuscripts within each category (neurotechnology and AI vs. other).
**Table S2.** Inter‐rater agreement of reviewer comment classification.
**Figure S2.** Number of reviewers assessing neurotechnology/AI versus other studies.
**Table S3.** Supplement to Table 2: Recommendations for reporting neurotechnology and artificial intelligence studies. Includes examples from reviewer comments.

## Data Availability

The data supporting the findings of this study contain potentially identifying or sensitive information and, therefore, cannot be made publicly available.
