# Supplementary material for: How to report neurotechnology and artificial intelligence studies in epilepsy: Peer‐review‐inspired recommendations
Source: Epilepsia Open. 2025 Nov 27:10.1002/epi4.70194. Online ahead of print. doi: 10.1002/epi4.70194 (PMC13394119; doi:10.1002/epi4.70194)
Supplement: Supplementary file 1 — Table S1. Peer reviewer comment classification checklist (adapted from van Lent et al., BMJ Open 2015). Figure S1. Number of accepted versus rejected manuscripts within each category (neurotechnology and AI vs. other). Table S2. Inter‐rater agreement of reviewer comment classification. Figure S2. Number of reviewers assessing neurotechnology/AI versus other studies. Table S3. Supplement to Table 2: Recommendations for reporting neurotechnology and artificial intelligence studies. Includes examples from reviewer comments. [file EPI4-9999-0-s001.docx]

# Supplementary Table 1

Peer Reviewer Comment Classification Checklist (adapted from Van Lent M. et al, BMJ Open 2015)

| Importance | Research question and results of limited clinical relevance/narrow interest |
| --- | --- |
| Originality | Lack of novelty; paper does not add much to what is already in the published literature |
| Abstract | Discrepancies between data reported in abstract and main text of the paper |
|  | Abstract does not correctly reflect the paper; information in abstract incomplete/inaccurate |
| Background | Incorrect/missing background information in introduction |
|  | Poor justification for conducting the study; insufficient problem statement/rationale provided |
| Methods | Methods inadequately reported; trial design, participants, interventions, outcome measures used, sample size calculation, randomisation, or blinding inaccurately described |
|  | Poor experimental design; methodology/study population/data collection methods flawed or questionable |
|  | Statistical analysis methods inappropriate or inadequately reported |
| Results | Flow of participants through the study unclear; no explanations provided for non-randomized/excluded subjects or drop-outs |
|  | Results not answering study question, study outcome data incomplete, results inaccurately presented |
| Discussion | Conclusions inappropriate in relation to study design or results, or overinterpretation of results |
|  | Study insufficiently related to prior work in the literature; not discussed how study results support or disagree with previous research |
|  | Limitations/shortcomings of the study not acknowledged or not sufficiently discussed how they might affect the study results and their interpretation |
|  | Meaning of study results inadequately discussed; potential explanations and implications insufficiently addressed in discussion |
| References | Errors in reference citation; references not appropriate for statements they are meant to support |
|  | References missing in article, or outdated/irrelevant references used |
| Presentation | Inaccurate/absent/incomplete tables or figures, discrepancies between tables, or discrepancies between main text and tables |
|  | Title not representative of the study |
|  | Poor writing; typos, unclear language, incorrect terminology, text difficult to follow, wordiness, incorrect use of sections in paper |
| Ethics | Not clear which research ethics committee provided approval for the study |
|  | Informed consent procedures inadequately reported, or other ethical issues related to the study |
| Study Registration | Deviations from the trial registry or protocol; not adequately explained why these deviations occurred |
|  | Trial registration absent/incomplete or registration number missing, study protocol not published/provided by authors, or CONSORT statement/diagram missing |
| COI | Potential bias introduced by author conflicts of interest, or funders contribution to the study unclear |
|  | Systematic bias or spin in the interpretation of results in favour of the study sponsor |

# Supplementary Figure 1

Acceptance Rate

|  | Accept | Reject |
| --- | --- | --- |
| Neurotechnology & AI | 44 | 23 |
| Other | 183 | 79 |

Number of accepted vs. rejected manuscripts within each category (Neurotechnology and AI vs. Other)

# Supplementary Table 2

Inter-rater agreement of reviewer comment classification

| **Main themes** | **% Agreement** | **Subthemes** | **% Agreement** |
| --- | --- | --- | --- |
| Importance | 87% | Research Question of Limited Relevance | 100% |
| Originality | 100% | Lack of Novelty | 100% |
| Abstract | 87% | Discrepancies between abstract and main text | 100% |
|  |  | Abstract does not correctly reflect paper | 87% |
| Background | 60% | Incorrect/missing background information | 73% |
|  |  | Poor justification/rationale for conducting study | 60% |
| Methods | 73% | Methods inadequately reported | 87% |
|  |  | Poor experimental design | 87% |
|  |  | Statistical analysis methods inappropriate | 60% |
| Results | 100% | Flow of participants through study unclear | 73% |
|  |  | Study outcome data incomplete | 60% |
| Discussion | 100% | Conclusions inappropriate / overinterpretation | 60% |
|  |  | Study insufficiently related to literature | 73% |
|  |  | Limitations not sufficiently discussed | 87% |
|  |  | Meaning results inadequately discussed | 73% |
| References | 87% | Errors in reference citation | 87% |
|  |  | References missing / irrelevant references used | 73% |
| Presentation | 100% | Inaccurate tables or figures | 100% |
|  |  | Title not representative of the study | 100% |
|  |  | Poor writing | 100% |
| Ethics | 87% | Ethics committee approval not clear | 100% |
|  |  | Other ethical issues related to study | 87% |
| Study Registration | 100% | Deviations from registry or protocol | 100% |
|  |  | Registration/protocol missing | 100% |
| COI | 100% | Bias by author COIs / contribution funder unclear | 100% |
|  |  | Systematic bias or spin in favour of sponsor | 100% |
| Overall Agreement = 88% | | | |

# Supplementary Figure 2

Number of Reviewers

|  | Neurotechnology & AI | Other |
| --- | --- | --- |
| 1 | 14 | 43 |
| 2 | 49 | 194 |
| 3 | 3 | 23 |
| 4 | 1 | 2 |

Number of reviewers assessing Neurotechnology/AI vs. other studies.

# Supplementary Table 3

Supplement to Table 2: Recommendations for reporting neurotechnology and artificial intelligence studies. Includes examples from reviewer comments.

| **Theme** | **Recommendation(s)** | **Example pitfalls in epilepsy^#^** | **Example(s) from reviewer comments*** |
| --- | --- | --- | --- |
| **Background and Objectives** | | | |
| Justify the use of the new technology or AI | Justify the choice of the new technology or AI method. Describe how it adds to the existing clinical workflow or pathway. Compare it with current existing similar solutions. Simpler methods are preferred if performance is comparable. Avoid using new technology or AI solely for novelty. | An AI-based seizure detection algorithm requiring high computational resources has similar performance to a simpler algorithm based on a small number of features. | "(…) the authors need to describe the clinical relevance of this technology explicitly. "  "This information per se does not add to the clinical workup (…) when any clinician would immediately look for (…)"  "The primary indication to advocate this as an appropriate modality of investigation (...) is if this influences prognosis (as indeed is the case with (...). This paper does not clearly establish this and this needs to be justified." “It is unclear why this layer of complexity to the (...) needs to be considered when they offer virtually no improvement.” “(…) feature extraction techniques have been used by other (…) studies. The best-performing approach (...) is identical to techniques used by other studies when extracting features.” |
| Align study goals with the analysis | Clearly define study objectives and ensure analysis aligns with these goals. Provide rationale for the study hypotheses. | A study on a seizure detection system aimed at providing a real-time alarm to patients does not report on the detection latency of their algorithm. | "The authors claim that use of (…) can improve retention rate and reduce the occurrence of side effects in the conclusion. How was this assessed?" |
| **Methods** | | | |
| Study Population / Data source | Describe inclusion and exclusion criteria for participants, screening method(s). Describe all data sources used for model training and testing, including type, representativeness, and rationale. Consider selection bias. | A post-operative outcome algorithm is trained on a single centre with no experience in a particular surgical technique. The model is unlikely to generalise to other centres. | "In a line or two explain skewed sex ratio in (...) group" "As they retrospectively enrolled patients with MRI scanning in the clinical practice, there could possibly exist selection bias" |
| Participant / Data flow | Report participant flow, follow-up or loss to follow-up. Describe handling of missing or poor-quality data, including imputation or exclusion procedures. | In a neurostimulation trial, patients with device complications or non-effectiveness may drop out early and less likely attend monitoring visits, leading to an inflated estimation of device effectiveness. | "What was the follow-up duration of patients?" "Who recruited healthy controls?" "At what time intervals were patients instructed to enter data and how often was this reviewed by the responsible parties?" |
| Description of the technology | Describe the technology fairly for readers who might not be familiar with it. | A paper describes a "wearable seizure detector" but does not clarify that the device only relies on accelerometery, and is only useful to detect major motor seizures. | "Additionally, there is lack of a detailed description of the (...) equipment, such as the number of electrodes or channels used for recording." |
| Control group or intervention | Aim to isolate the effect of the new technology or method, by comparing with a control group or intervention. | A novel neurostimulation device is associated with a reduction in seizure frequency, however there is no sham control period to isolate the effect of stimulation from the effect of surgery. | "Is the control group a sham-operated group?" "The lack of healthy controls to serve as a true baseline for (...) is a limitation." "The authors claim that use of (…) can improve retention rate and reduce the occurrence of side effects in the conclusion. How was this assessed? Was there a control group of patients that did not use the app with poorer outcomes?" |
| Data collection and preprocessing | Describe them enough to allow replication. Justify the choice of preprocessing steps or stimulation parameters with previous literature or available recommendations  - EEG studies: e.g. sampling rate, filtering, referencing, montages, artefact rejection, windows, electrode material, data normalization  - Neuroimaging studies: e.g. type of scanner, consistency across patients, timing/duration of tracer uptake, circumstances of scanning | In a neuroimaging AI lesion detection study, two centres use different scanner. One centre has disproportionally more lesional cases than the other. The model learns scanner-specific features, not pathology. A study aimed at detecting high frequency oscillations uses inappropriate high pass filtering. | "Did the authors account for a potential effect of hospital site? Are there reasons to expect differences in model performance between sites, for example, if they use different note styles, or have different patient populations?" |
| Data annotation | Use qualified data annotators or raters, ideally blinded for study groups or outcomes. Use standard or validated data collection instruments. Acknowledge limitations in reliability of data annotations | A non-qualified PhD student reviews EEG data to annotate seizure patterns and semiology, leading to many falsely annotated events (artefacts) as seizures. A study uses information from a seizure diary smartphone application, where the diagnosis of epilepsy is not confirmed by experts. | "The (...) are screening instruments. I would not base the diagnosis of (..) on these results (…)." "Please elaborate on the experience of the experts analyzing the EEG recordings." "Automatic sleep staging: Was the scoring cross-checked by a epileptologist? Epileptic activity can make sleep scoring more challenging. Was this automated approach used in patients with epilepsy before?" "How many reviewers evaluated the images and provided classifications? And when there were disagreements about pattern classification, how were they resolved? Are the reviewers blinded to the post-surgical outcomes?" |
| Separate training from testing datasets | Use separate datasets for training and testing. Describe partitioning, cross-validation, or bootstrapping methods. Only tune model parameters on the training or validation datasets, not on the testing dataset | In a seizure forecasting study, the calculation of the predictor features is influenced by future datapoints. | "A replication cohort to validate the findings of the multivariate analysis should have been included in the study design." "Also, if they based their training and test sets on the same sample (…) there is a high chance that their model is overfitted." "Authors mentioned that the analysis was performed on data from (...) patients from the (center name). Please clarify why data from the other 2 centers was excluded. Wouldn’t it be interesting to evaluate the performance across a larger cohort of patients and assess if performances are stable on an independent dataset (from another center) ?" "Are the RR intervals for the entire recording calculated at the start? If so this will likely break the pseudo-prospective nature of the study" "The authors have not provided a hold-out dataset, therefore it remains unknown how well the results that they report can generalize." |
| Sample size | Perform and report sample size estimation / power calculation, adequate for the objectives of the study, or justifiy low sample size, or use appropriate methods to circumvent the small sample size (e.g. transfer learning from another dataset). | In a neuroimaging study investigating lesion detection in epilepsy, training a neural network with thousands of parameters on only a small cohort of patients can lead to overfitting, causing artificially high performance on the training data but poor generalization to new patients. | "My main concern in this study is low sample size and lack of power analysis" |
| Class Imbalance | Address or correct for class imbalance. Consider balanced sampling when splitting data into training and testing | In a seizure detection study, an algorithm that classifies all events as "interictal" may have 99% accuracy, simply because the ratio of interictal to ictal samples is large. | "There is an issue with the way the authors addressed the imbalanced number of (...) controls and (...) patients."  "Give significant class imbalance, a precision-recall curve may be more appropriate." |
| Model Specification | Describe the AI models - input structure, architecture, output structure. Specify model type, approach, and all steps in building, including hyperparameter tuning, parameter handling, and internal validation. | A model claims to predict seizures from EEG but does not specify whether the input is raw EEG, power spectral features, or time-of-day information. | "What was the specific input for the NLP analysis?" "which begs the question of how exactly they thresholded the maps while achieving the reported statistical significance level." "We are not given any information on what variables were entered in the model, other than the fact that they added connectivity strength for specific region pairs. Were any other variables entered in the regression?" |
| Reference standard | Use an appropriate clinical reference standard when comparing with your solution. Comparison between the proposed solution and clinical standard should be direct and fair (e.g. manual vs. automated methods, old vs. new technology). | A seizure detection study compares an automated seizure detection algorithm with manual review. However, experts only review the algorithm's detections, and therefore there is no assessment of false negatives.  A seizure detection study compares a detection algorithm with video analysis, including for seizures with no clear clinical manifestations. | "The gold standard bears several important limitations. First, only (...) was used to establish the gold standard. While this is sufficient for (...) it is insufficient for (...)." "I worry about a bias toward higher inter-rater reliability for the (...) results relative to (...) due to the fact that one physician performed (...), but (...) was performed externally." |
| Multiple comparisons | Address for multiple comparisons and false discovery rate. | A study of high-density EEG spectral analysis computes spectral power in four frequency bands, per channel, leading to hundreds of statistical tests. | "How did the authors correct for multiple comparisons when deriving the maps?" "No multiple-comparison correction is applied to the alpha value despite multiple models being used per patient. Please consider including a Bonferroni correction, or similar." "It is helpful to clarify whether a correction for multiple comparisons was employed. If not, the authors want to discuss the usage of uncorrected p-values as a limitation." |
| **Results** | | | |
| Model evaluation and comparison | Use clinically relevant performance metrics. Report effect sizes and measures of dispersion (e.g. confidence intervals) around performance. Consider performing feature importance analyses. Discuss proxy outcomes and limitations. | In a post-surgical outcome prediction study, only the AUC is provided, whilst the negative predictive values (NPV) would likely have more clinical value. | "The spectral component as well as temporal dynamics of these different seizures could be extremely variable and thus affect classification performances. Did the authors consider this information somehow?" "The authors reported AUC without NPV. Whereas there are plenty of reasons to use AUC as a general purpose check for accuracy, in this particular clinical scenario, NPV is of critical importance." "I question the authors’ interpretation that the (...) model should be held up as the “best performing” model, and I wonder if a more statistically appropriate interpretation would be that all three models performed similarly." "Is the model predicting seizure freedom, or predicting a procedure with better outcomes?" "Why did the author apply (...) as indicators of the efficacy of (...) treatment?" |
| Model Fairness | Consider whether the model is fair or whether there is a risk of perpetuating existing biases in the dataset. | A post-operative outcome model uses age as a major determinant of poor outcome, potentially leading to self-fulfilling prophecy of denying surgery to older individuals. | "The presence of “his” and “he” in this list of overfitting words raises concern for a gender bias (...) if the model is preferentially identifying male patients." |
| Technology acceptability and safety | Describe how relevant stakeholders (patients, clinicians, caregivers) were involved in technology and AI design and conceptualization. Report acceptability and usability of the new technology or method, e.g. adherence, app usage. Report safety of using the technology. | A wearable device company develops an epilepsy monitoring device with a poor design, which leads to a low acceptability and high attrition rate from users. | "Was there any attempt to assess how often and reliably the app was used?" "Additional details about how (…) was used by participants during the study would be helpful, as this appears to be the main novelty in the current study." "How did this compare to patients who did not use the app?" "Adverse events were barely mentioned." "Do you have information about the lag-time between invitation to the tools and first use of the tools? Do you have information about number of log-ins and/or amount of time spent interacting with the tools?" |
| Confounding | Address or acknowledge confounding | In an EEG study developing an AI algorithm to diagnose epilepsy, using patient recordings on anti-seizure medication while controls are medication-free may cause the model to learn drug-induced EEG changes rather than true disease-related features. | "Are patients who have more seizures more interested in device?" "EEG (...) can be contaminated by EMG signals from facial, temporal, and ocular muscles. These signal deflections can affect the connectivity measures of interest." "If patients received different medications during this study, how we can make sure that the observed effects are caused by (...)?" "Please indicate if patients were currently on any medications that can affect cognitive abilities (e.g., topiramate, barbiturates, benzodiazepine)." "The model could be differentiating the procedure rather than the characteristics that portend surgical outcome." |
| **Discussion** | | | |
| Clinical applicability and integration | Describe the technology integration into clinical workflow, including technical requirements, human interaction, and user expertise. Discuss technology limitations, e.g. cost, technical support, data security, limited adherence, device deficiency. Describe the target population for which the technology could be useful. Provide future directions for further research and validation of the technology. | A seizure monitoring device placed under the mattress is only useful when patients are in bed. A neuroimaging automated lesion detection solution uses a research-grade, non-medically approved preprocessing toolbox. | "the authors should give or discuss how much computation resources and time are needed using (…) and how much improvement compared to other (…) in the literature" "Could the authors elaborate a bit more on their opinion regarding the applicability and clinical place of their method knowing that its performance is weaker than (…) analysis." "As you appropriately pointed out, it is difficult to reliably make medical decisions based on the data collected from the device without (...) verification." "For (...) a highly reputed Matlab toolbox is used. It should be discussed how the use of a toolbox aiming at research customers is suited for the use in a clinical workflow." "Could the authors provide an estimate of how many patients benefit from (...) this device?" |
| **Presentation** | | | |
| Terminology | Use updated, accepted or widely used terminology. Refer to glossaries, recent guidelines, ILAE position papers and journal instructions for authors. Avoid technical jargon and adapt the language to the target journal. | In a study on automated EEG seizure detection, the authors use the term 'epileptic events' interchangeably with 'seizures' and 'spikes', without defining each; this inconsistency confuses readers about whether the model detects ictal episodes, interictal discharges, or both. | "Nomenclature, clinical details: what is the meaning of sBTCS ? do the authors mean FBTCS? please clarify/use the ILAE-recommended terminology." "The 300-400 words spent on standard description of machine learning models can be trimmed to a shorter paragraph. The descriptions of machine learning models such as SVM, Decision Trees, Random Forest, and LSTM can be condensed." |
| **Ethics** | | | |
| Fairness | Avoid writing with bias, e.g. commercial bias. Be fair in the interpretation of your results. | Authors affiliated with a neurotechnology company emphasize the "superior performance" of their proprietary software without disclosing that competing open-source models were not evaluated under identical conditions. | "It would be important to list authors conflict of interest" "It isn’t clear to me if Dr (...) should declare a conflict of interest. (...) appears to work for (...)." "Moreover, the paper is apparently written with bias" |
| Data availability and code availability | Ensure transparency and reproducibility by sharing code, models, and documentation. | In an AI study on seizure detection, not sharing preprocessing code led to an undetected labelling error where postictal segments were included as seizures, artificially inflating model performance. | "Will the code be shared publicly or made available?" |

^#^These are hypothetical examples and not directly related to any real examples in the dataset. * Examples edited to remove content that could potentially identify a specific study or authors.
